# Non‐coding RNA MFI2‐AS1 promotes colorectal cancer cell proliferation, migration and invasion through miR‐574‐5p/MYCBP axis

**DOI:** 10.1111/cpr.12632

**Published:** 2019-05-16

**Authors:** Chenglong Li, Fengbo Tan, Qian Pei, Zhongyi Zhou, Yuan Zhou, Lunqiang Zhang, Dan Wang, Haiping Pei

**Affiliations:** ^1^ Department of Gastrointestinal Surgery, Xiangya Hospital Central South University Changsha China

**Keywords:** colorectal cancer, LncRNA MFI2‐AS1, miR‐574‐5p, MYC binding protein

## Abstract

**Objective:**

Long non‐coding RNAs (lncRNAs) and microRNAs (miRNAs) play essential roles in the tumour progression. LncRNAs mostly act as competing endogenous RNAs (ceRNAs) by sponging miRNAs. This study aimed to study the association of a novel lncRNA MFI2‐AS1 with miR‐574‐5p/MYCBP axis in the development of colorectal cancer (CRC).

**Methods:**

Ninety‐four CRC tissues and paired adjacent non‐tumour tissues were included in our study. The relative expression level of MFI2‐AS1 was detected, and its relationship with clinico‐pathological factors was analysed. Then, the CRC cells lines (LoVo and RKO) were transfected with MFI2‐AS1 siRNA, miR‐574‐5p mimics and inhibitors. Cell proliferation, migration, invasion, cell cycle distribution and DNA damage in response to different transfection conditions were examined. Dual‐luciferase reporter assay was performed to identify the target interactions between MFI2‐AS1 and miR‐574‐5p, miR‐574‐5p and MYCBP.

**Results:**

LncRNA MFI2‐AS1 and MYCBP were up‐regulated in CRC tissues when compared with adjacent non‐tumour tissues. The expression levels of MFI2‐AS1 were significantly associated with tumour histological grade, lymph and distant metastasis, TNM stage and vascular invasion. Both MFI2‐AS1 siRNA and miR‐574‐5p mimics inhibited proliferation, migration and invasion in LoVo and RKO cells. The transfection of miR‐574‐5p inhibitor showed MFI2‐AS1 siRNA‐induced changes in CRC cells. Dual‐luciferase reporter assay revealed target interactions between MFI2‐AS1 and miR‐574‐5p, miR‐574‐5p and MYCBP.

**Conclusions:**

These findings suggested that lncRNA MFI2‐AS1 and MYCBP have promoting effects in CRC tissues. LncRNA MFI2‐AS1 promoted CRC cell proliferation, migration and invasion through activating MYCBP and by sponging miR‐574‐5p.

## 1 INTRODUCTION

Increasing evidences show the crucial roles of genetic and epigenetic dysregulation in the generation and development of tumours.^1^, ^2^, ^3^ Recently, the multifunctional roles of non‐coding RNAs, including microRNA (miRNA) and long non‐coding RNA (lncRNA), have been identified, which are deeply interested and extensively studied by the researchers.

Long non‐coding RNAs are important and play diverse roles in regulating genetic transcription,^4^ modulating embryonic and neoplastic differentiation,^5^, ^6^ pathogenesis of multiple diseases^7^, ^8^ and drug resistance in tumour cells.^9^, ^10^, ^11^ LncRNA by itself cannot regulate these biological processes. The most widely recognized theory is that the lncRNA acts as a competing endogenous RNA (ceRNA) by decoying miRNAs to regulate the expression of miRNA's target genes and change the level of transcription and translation products. In addition, some lncRNAs have similar structure to mRNAs, including a polyA tail and promoter region. The latter empowers the lncRNAs to manage the transcription of its downstream genes directly.

Theoretically, in ceRNA, lncRNA acts as a miRNA sponge or trapper to modulate miRNA‐mRNA axis‐mediated biological processes. In the field of cancer, there are countless studies that show the regulation of lncRNAs in cancer pathogenesis, development, metastasis and prognosis. For instance, the widely studied lncRNAs, including MALAT1, H19 and HOTAIR, have been reported to promote tumour aggressiveness.^12^, ^13^, ^14^, ^15^ The expression of lncRNA HOTAIR in epithelial cancer cells increased breast cancer invasiveness and metastasis via enforcing the expression of polycomb repressive complex 2^14^; and MALAT1 expression promotes the aggressiveness of renal cell carcinoma (RCC).^12^ These data suggested the important roles of lncRNAs in tumorigenesis and development.

Colorectal cancer (CRC) is a common cause of cancer‐related deaths worldwide with high morbidity, especially in the older population.^16^, ^17^ The 5‐year survival rate of patients with CRC varied in stages, and at stage IV or metastatic stage is approximately 10%.^18^ There are several studies that discussed the association of lncRNA dysregulation with the occurrence and progression of CRC.^19^, ^20^, ^21^, ^22^, ^23^ These studies demonstrated the complex mechanism of CRC pathogenesis by lncRNAs.

According to a recent study, the lncRNA MFI2‐AS1 (chr3:196729777‐196731615) has been identified and was up‐regulated in pancreatic cancer cells and sporadic localized clear‐cell RCC (ccRCC).^24^, ^25^ Flippot et al showed that the RNA MFI2‐AS1 expression was strongly associated with the recurrence and poor disease‐free survival (DFS) of patients with sporadic localized clear‐cell RCC.^25^ Using bioinformatics analysis, we predicted that MFI2‐AS1 sponged miR‐574‐5p, a candidate oncogene that play multifunctional roles in cancer metastasis, including thyroid carcinoma^26^, ^27^ and CRC.^28^ However, no study suggested the association of lncRNA MFI2‐AS1/miR‐574‐5p axis with the tumorigenesis or progression of CRC. Hence, in order to investigate this, we detected the expression of lncRNA MFI2‐AS1 in CRC tissues and in vitro experiments were performed to assess the association of potent lncRNA MFI2‐AS1/miR‐574‐5p axis with CRC development.

## 2 MATERIALS AND METHODS

### 2.1 Clinical specimens

A total of 94 CRC tissues and paired adjacent non‐tumour tissues were collected from patients undergoing surgical resection from January 2013 to June 2014 at Xiangya Hospital, Central South University. None of them had received radiotherapy or chemotherapy prior to surgical resection. All isolated samples were snap frozen and then stored at −80°C before RNA extraction. Our experimental protocol was approved by the Ethics Committee of Central South University. Written informed consent was obtained from all participants.

### 2.2 Data mining

The data of the expression of MFI2‐AS1 in CRC were acquired from the Gene Expression Profiling Interactive Analysis (GEPIA) (http://gepia.cancer-pku.cn/), a bioinformatics database based on TCGA and GTEx, facilitating various online analysis including differential expression analysis, box plotting, patient survival analysis and so on. The relevance of DFS and overall survival (OS) rates with the expression of MFI2‐AS1 was also analysed by the GEPIA online database.^29^

### 2.3 Cell lines and culture conditions

An immortalized colonic epithelial cell line (FHC) and five human CRC cell lines (HT29, LoVo, HCT116, SW480 and RKO) were obtained from ATCC. All cells were cultured in RPMI 1640 medium (Invitrogen), with 10% FBS (Hyclone) and 1% penicillin‐streptomycin (Hyclone) at 37°C in 5% CO_2_.

### 2.4 Fluorescence in situ hybridization

The in situ expression of lncRNA MFI2‐AS1 was examined using RNA fluorescence in situ hybridization (FISH) staining as previously described. The fluorescent RNA FISH probe (dye on the 3′ end, coupled with Alexa Fluor 594) was purchased from Genechem Co. Ltd. (China). The cells were then fixed on slides using 4% paraformaldehyde and incubated with probes (50 nmol/L) at 37°C overnight. Cells were then counterstained with propidium iodide (PI). Digital fluorescent photographs were captured by using a fluorescent microscope (Olympus).

### 2.5 Cell transfections

Small interfering RNA (siRNA) that directly target MFI2‐AS1, scrambled oligonucleotides, miR‐574‐5p mimics, inhibitors and scrambled sequences were purchased from Genechem Co. Ltd. Cell transfections were performed using Lipofectamine 2000 reagent (Invitrogen). All experiments were performed in triplicates. All cells were incubated in RPMI 1640 medium at 37°C in 5% CO_2_ for 72 hours.

### 2.6 Total RNA isolation and quantitative real‐time PCR

Trizol reagent (TaKaRa) was used to extract total RNA from tissue samples and cultured cells. The first cDNA strand was synthesized according to the methods provided by Bestar qPCR RT kit (DBI Bioscience) and amplified using specific primers (Invitrogen) listed in Table 1. Quantitative real‐time PCR (qRT‐PCR) analysis was performed to determine the relative expression level of lncRNA and miRNA according to the following conditions: 94°C for 2 minutes, followed with 40 cycles of 94°C for 20 seconds, 58°C for 20 seconds, 72°C for 20 seconds and finally extended at 72°C for 4 minutes. Amplification was implemented using a Bestar^®^ Sybr Green qPCR master mix kit (DBI Bioscience) on Agilent Stratagene Mx3000P RT‐PCR machine (Agilent Technologies). The relative expression level of detected genes was determined using the 2^−∆∆Ct^ methods. GAPDH and U6 gene were used as internal reference genes for lncRNA and miRNA, respectively.

**Table 1:** The PCR primers used in this study

Gene name	Primers	Sequence (5′‐3′)
GAPDH	Forward	TGTTCGTCATGGGTGTGAAC
	Reverse	ATGGCATGGACTGTGGTCAT
MFI2‐AS1	Forward	TACATACAGTGACCCAAAGAGCA
	Reverse	CAGTGCTTCTGAACGCCTCTT
U6	Forward	CTCGCTTCGGCAGCACA
	Reverse	AACGCTTCACGAATTTGCGT
miR‐574‐5p	RT	CTCAACTGGTGTCGTGGA GTCGGCAATTCAG TTGAGAC ACACTCA
miR‐574‐5p	All R	CTCAACTGGTGTCGTGGA
miR‐574‐5p	Forward	ACACTCCAGCTGGG TGAGTGTGTGTGTGTGA
miR‐19‐3p	RT	CTCAACTGGTGTCGTGGA GTCGGCAATTCAG TTGAGAC TCAGTTT
miR‐19‐3p	All R	CTCAACTGGTGTCGTGGA
miR‐19‐3p	Forward	ACACTCCAGCTGGG TGTGCAAATCTATGCA AAA
miR‐218‐5p	RT	CTCAACTGGTGTCGTGGA GTCGGCAATTCAG TTGAGAC ACATGGT
miR‐218‐5p	All R	CTCAACTGGTGTCGTGGA
miR‐218‐5p	Forward	ACACTCCAGCTGGG TTGTGCTTGATCTA ACC
miR‐375	RT	CTCAACTGGTGTCGTGGA GTCGGCAATTCAG TTGAGAC TCACGCG
miR‐375	All R	CTCAACTGGTGTCGTGGA
miR‐375	Forward	ACACTCCAGCTGGG TTTGTTCGTTCGGCTCGC
miR‐130‐3p	RT	CTCAACTGGTGTCGTGGA GTCGGCAATTCAG TTGAGAC ATGCCCT
miR‐130‐3p	All R	CTCAACTGGTGTCGTGGA
miR‐130‐3p	Forward	ACACTCCAGCTGGG CAGTGCAATGTTAAA AGG

### 2.7 Cell counting kit‐8 assay

Transfected cell viability was detected using Cell counting kit‐8 (CCK‐8; Beyotime Institute of Biotechnology) according to the manufacturers' instructions. In brief, the cells were seeded into 96‐well plates at a final density of 5 × 10^3 ^cells per well and then transfected in different conditions. At 24, 48 and 72 hours post‐transfection, the cells were further incubated with 20 μL per well of CCK‐8 solution for 2 hours. Then, the absorbance at 450 nm (OD_450nm_) was measured. Each experiment was performed in triplicate.

### 2.8 In vitro transwell assay

Cell invasion ability was detected using 24‐well invasion chambers (Coring) coated with Matrigel (BD Biosciences). The upper chamber was supplemented with serum‐free RPMI 1640 medium and 1 × 10^5 ^CRC cells, and the lower chamber was filled with full RPMI 1640 medium supplemented with 10% FBS (Hyclone). The chambers were then maintained at 37°C in 5% CO_2_ for 48 hours. Subsequently, the cells that were adhered to the undersurface of the filter membranes were removed, and the invaded cells were then fixed and stained with crystal violet for cell counting. An olympus microscope (CX41) was used to capture the digital photographs at five arbitrarily selected (non‐overlapped) fields. Then the average number of the invaded cells was counted.

### 2.9 Wound healing assay

Cell migration was detected using in vitro wound healing assay. Cells were placed into 24‐well plates and incubated for 24 hours for the formation of monolayer on the bottom plate. After that, a straight line was scratched onto the monolayer using a 200 μL micropipette tip. After 48 hours, the wound width was analysed using Axio Observer, the microscope (Carl Zeiss AG). All experiments were performed in triplicates.

### 2.10 Flow cytometric analysis

Cell cycle distribution was detected using flow cytometry. Cells were transfected under different conditions for 48 hours and then were harvested and fixed. For cell cycle distribution, cells were incubated with PI for 20 minutes. Then, the cell cycle distribution was analysed using the BD FACS Calibur flow cytometry (BD Biosciences).

### 2.11 Hoechst fluorescent staining

Hoechst fluorescent staining was performed to detect DNA damage and cell apoptosis. In brief, the cells were plated in 96‐well plate and then incubated with 1 μg/mL Hoechst 33258 (Life Technologies) solution for 10 minutes at room temperature. The cells were then washed and examined using a Leica TCS‐SP2 confocal microscope (Leica).

### 2.12 Western blot analysis

The fold change of MYCBP protein in CRC tissues and transfected cells was detected using western blotting. Protein lysates were extracted from CRC tissues and cell lines using lysis buffer (Beyotime), followed by quantification and separation on 10% SDS‐PAGE (Invitrogen). The proteins were then immunoblotted onto the PVDF membrane (Millipore) by using a primary antibody for human MYCBP (1:1000; Abcam) and GAPDH (1:10 000; Abcam). HRP goat anti‐rabbit IgG (1:20 000; Boster Biotechnology) was then added as a secondary antibody. Enhanced chemiluminescence (ECL) system was used for visualizing bands.

### 2.13 Dual‐luciferase reporter assay

HEK293T cells were seeded in 24‐well plates and co‐transfected with miR‐574‐5p mimics/NC and pMIR luciferase reporter plasmids. Beforehand, plasmids were constructed into pMIR by inserting either wild‐type MFI2‐AS1, paired with the MFI2‐AS1 mutant binding site, or wild‐type 3′UTR of MYCBP, paired with mutant binding site. Then, plasmids were transfected into cells using Lipofectamine 2000 (Thermo Fisher Scientific) according to the manufacturer's protocol. At 48 hours after transfection, reporter luciferase activity was normalized to the control firefly luciferase activity by using the Dual‐Luciferase Reporter Assay System (Promega).

### 2.14 Statistical analyses

All statistical analyses were performed using GraphPad Prism 6.0 software. Data were expressed as mean ± standard deviation. Differences in the demographic characteristics between groups were analysed using unpaired *t* test or chi‐square test. *P* < 0.05 was considered to be statistically significant.

## 3 RESULTS

### 3.1 MFI2‐AS1 is up‐regulated in CRC tissues

**Figure 1:**
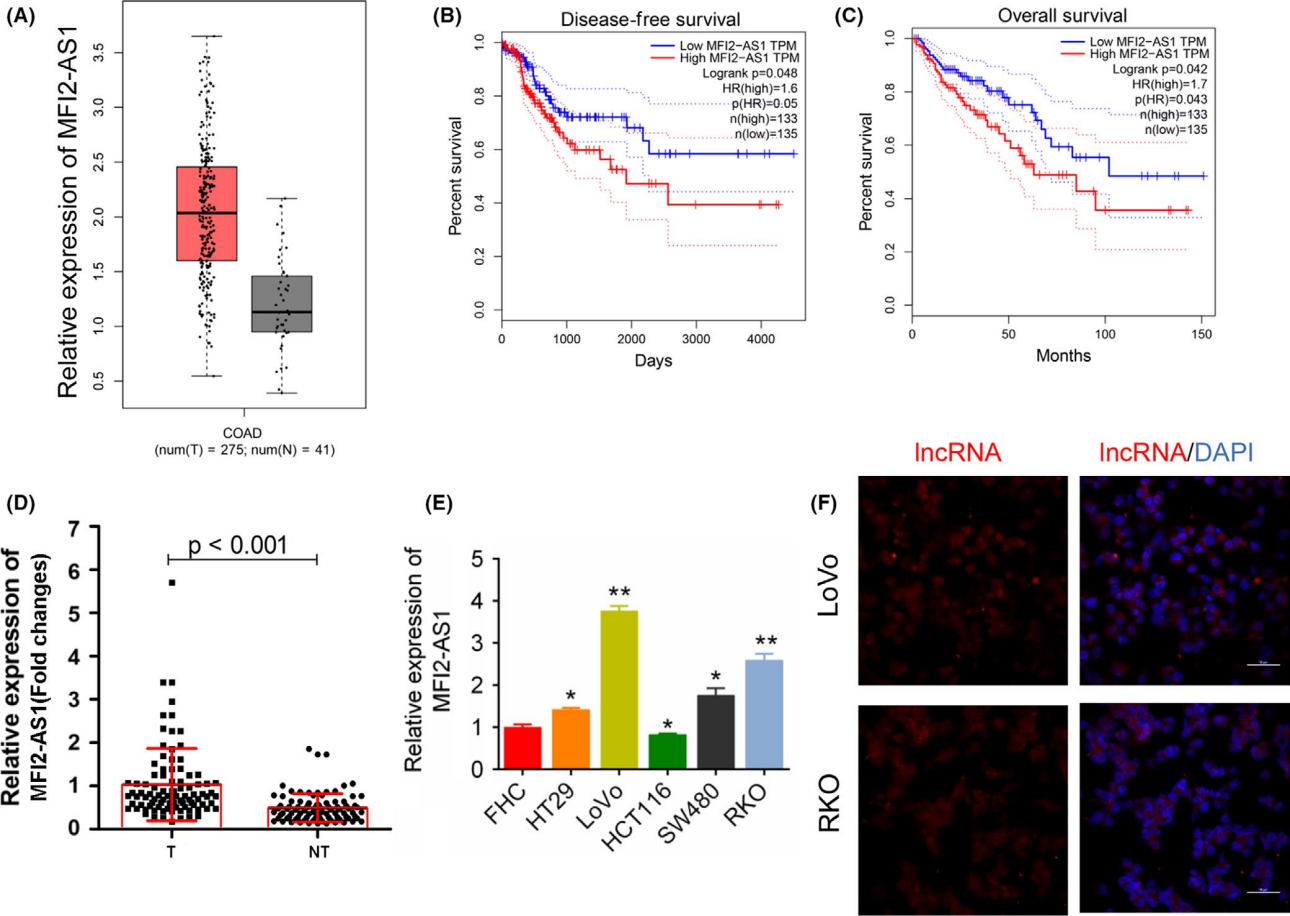
Expression of lncRNA MFI2‐AS1. A, from the GEPIA database, MFI2‐AS1 gene expression was significantly up‐regulated in CRC (n = 275) compared with corresponding normal tissues (n = 41). B and C, Kaplan‐Meier curves stratified by the expression level of MFI2‐AS1 in CRC showed a significant correlation with the expression level of MFI2‐AS1. The disease‐free survival and overall survival were computed by GEPIA. D, the relative expression level of lncRNA MFI2‐AS1 in tumour and adjacent non‐tumour tissues (n = 94, *P* < 0.001). E, the relative expression level of lncRNA MFI2‐AS1 in 5 human CRC cell lines. FHC was normal control. * and ** note *P* < 0.05 and *P* < 0.01 vs FHC, respectively. F, The fluorescence in situ hybridization of MFI2‐AS1 in CRC cells (Magnification, ×400, bar = 50 µm). NT, non‐tumour; T, tumour

The results of the box plots revealed that MFI2‐AS1 expression was significantly higher in CRC tissues by analysing the data form GEPIA (Figure 1A). The survival curves of CRC patients showed that the expression level of MFI2‐AS1 was significantly associated with DFS rate (*P* < 0.05; Figure 1B) and OS rate (*P* < 0.05; Figure 1C) by GEPIA. This revealed that high MFI2‐AS1 expression represented a poor prognosis, and MFI2‐AS1 might play a role in promoting the progression of CRC tissues. Moreover, we detected this in 94 CRC samples and confirmed that MFI2‐AS1 was markedly up‐regulated in CRC tissues compared with adjacent non‐tumour tissues (*P* < 0.001, Figure 1D). The up‐regulation of MFI2‐AS1 was observed in 4 of the 5 human CRC cell lines compared with normal control cell line FHC (*P* < 0.05), except HCT116 cell line, where its expression was down‐regulated (*P* < 0.05, Figure 1E). Moreover, we found that the expression of MFI2‐AS1 was related with several clinico‐pathological factors, and high MFI2‐AS1 was significantly correlated with tumour histological grade, lymph involvement, distant metastasis, TNM stage and vascular invasion (*P* < 0.05 for all, Table 2). There was no significant association found between MFI2‐AS1 expression and age, gender, T stage, pre‐operative serum CEA and CA 19‐9 levels, and the presence of perineural invasion (*P* > 0.05, Table 2).

**Table 2:** Correlation of MFI2‐AS1 expression with demographic characteristics of included CRC patients (n = 94)

Characters	N	Relative expression		
		Low	High	*P*‐value
Gender				
Male	54	26	28	0.6765
Female	40	21	19	
Age/Y				
≤60	47	25	22	0.5360
>60	47	22	25	
Histological grade				
High	32	21	11	0.0295
Middle or low	62	26	36	
T classification				
T1 + T2	10	6	4	0.5035
T3 + T4	84	41	43	
N classification				
N0	46	28	18	0.039
N1 + N2	48	19	29	
M classification				
M0	84	45	39	0.045
M1	10	2	8	
CEA				
<5 ng/mL	65	32	33	0.823
≥5 ng/mL	29	15	14	
CA 19‐9				
<35 KU/L	78	38	40	0.583
≥35 KU/L	16	9	7	
TNM stage				
I + II	45	28	17	0.023
III + IV	49	19	30	
Vascular invasion				
No	58	34	24	0.034
Yes	36	13	23	
Perineural invasion				
No	86	43	43	0.999
Yes	8	4	4	

Note

Low, fold change lower than 0.5. High, fold change larger than 0.5 (cut‐off = 2.71).

### 3.2 Inhibition of MFI2‐AS1 impedes CRC cell proliferation and metastasis

**Figure 2:**
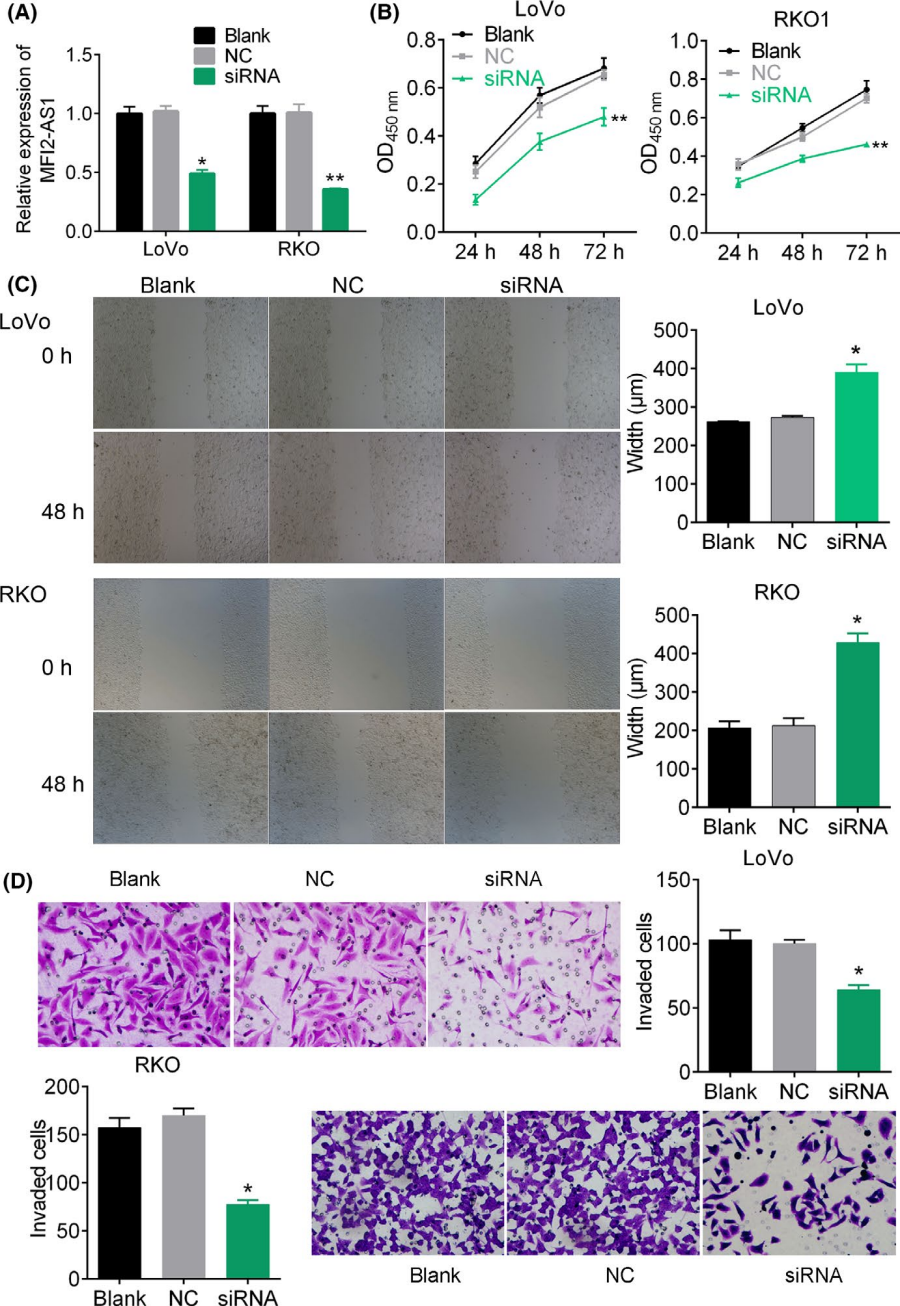
MFI2‐AS1 depletion impedes CRC cell proliferation and metastasis. A, the relative expression of MFI2‐AS1 in cells transfected with MFI2‐AS1 siRNA and corresponding negative control. B, cell viability analysis by CCK‐8 assay. C, wound healing assay. Cells are transfected with siRNA target MFI2‐AS1 for 48 h. D, cell invasion by transwell migration assay. Magnification, ×400. * and ** note *P* < 0.05 and *P* < 0.01 vs NC, respectively. NC, negative control

**Figure 3:**
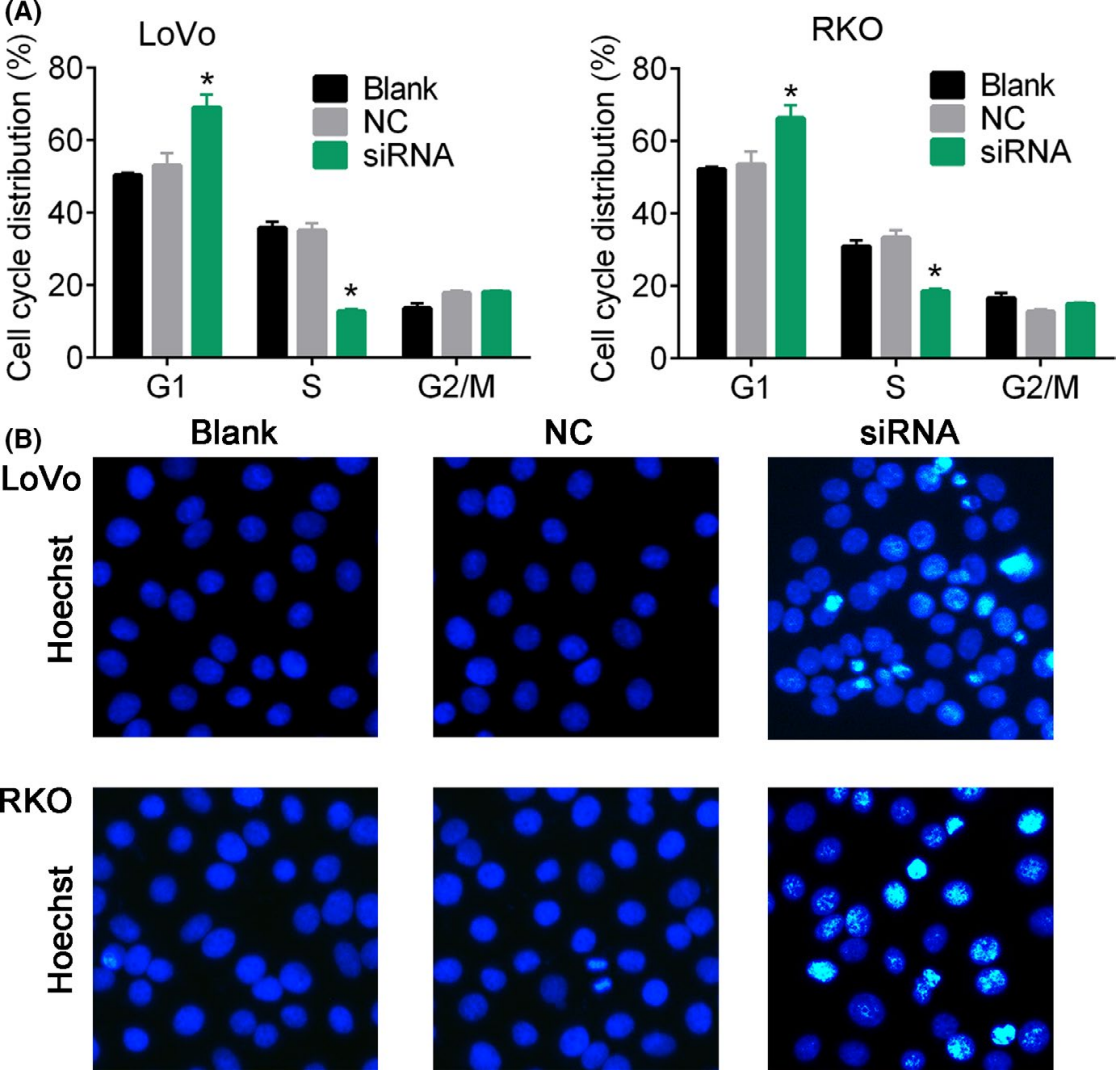
Cell cycle distribution and apoptosis analysis. A, cell cycle analysis was performed using flow cytometry. B, Hoechst staining for cells in response to siRNA. Cells were transfected with siRNA target MFI2‐AS1 for 48 h. * notes *P* < 0.05 vs NC. NC, negative control

Using FISH technique, we detected the expression of lncRNA MFI2‐AS1 in the cytoplasm of CRC cells (Figure 1F). In order to investigate whether the MFI2‐AS1 expression was associated with CRC development and metastasis, the CRC cell lines (LoVo and RKO) were transfected with siRNA target lncRNA MFI2‐AS1 (Figure 2A). The results showed that the inhibition of MFI2‐AS1 expression dramatically suppressed the cell viability (*P* < 0.01, Figure 2B), wound healing speed (*P* < 0.05, Figure 2C) and invasion of LoVo and RKO cells (*P* < 0.05, Figure 2D) compared with blank control. Further, flow cytometry analysis showed that the inhibition of lncRNA MFI2‐AS1 expression increased the percentage of cells at G1 stage and reduced the cells at S stage (Figure 3A and Figure S1). Hoechst 33258 staining showed that siRNA transfection increased Hoechst 33258‐positive cells in LoVo and RKO cells, respectively (Figure 3B). These data suggested that the MFI2‐AS1 inhibition suppressed CRC cell proliferation and invasion via arresting the cell cycle at G1 phage.

### 3.3 MiR‐574‐5p is a negative target of MFI2‐AS1

**Figure 4:**
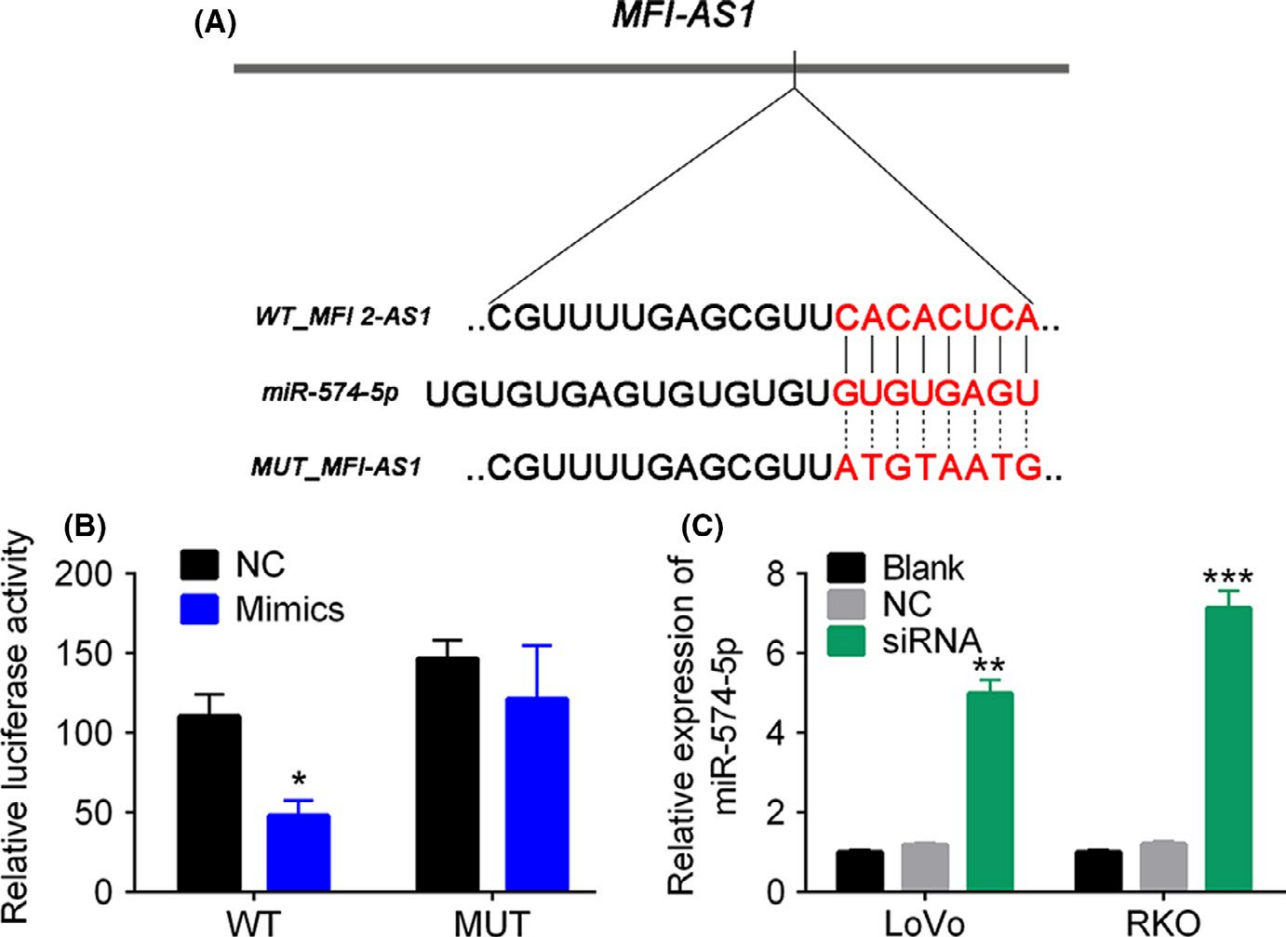
MFI2‐AS1 targets to miR‐574‐5p. A, the predicated bind sites of miR‐574‐5p to MFI2‐AS1. B, dual‐luciferase reporter assay. C, the relative expression level of miRNA in CRC cells transfected with MFI2‐AS1 siRNA. *, ** and *** note *P* < 0.05, *P* < 0.01 and *P* < 0.001 vs NC, respectively. MUT, mutant_MFI‐AS1, NC, negative control, WT, wild type

There are accumulating evidences showing that lncRNA regulates the biological processes by sponging miRNAs. In this study, a series of miRNAs were predicted be potential in sponging by MFS1‐AS1. We predicted that the miR‐574‐5p was a target of lncRNA MFI2‐AS1 by DIANA tools (Figure 4A) since the fold change in the expression of miR‐574‐5p was extraordinary higher than other miRNAs (Figure S2). Dual‐luciferase reporter assay showed that the administration of miR‐574‐5p mimic significantly reduced the relative luciferase intensity in 293T cells transfected with wild‐type 3′‐UTR sequence containing putative miRNA binding sites (*P* < 0.05, Figure 4B). In addition, the MFI2‐AS1 inhibition by siRNA significantly up‐regulated miR‐574‐5p in LoVo and RKO cells (*P* < 0.01, Figure 4C). These results suggested that miR‐574‐5p was a direct negative target of MFI2‐AS1.

### 3.4 MiR‐574‐5p suppresses CRC cell proliferation and metastasis by targeting MYCBP

**Figure 5:**
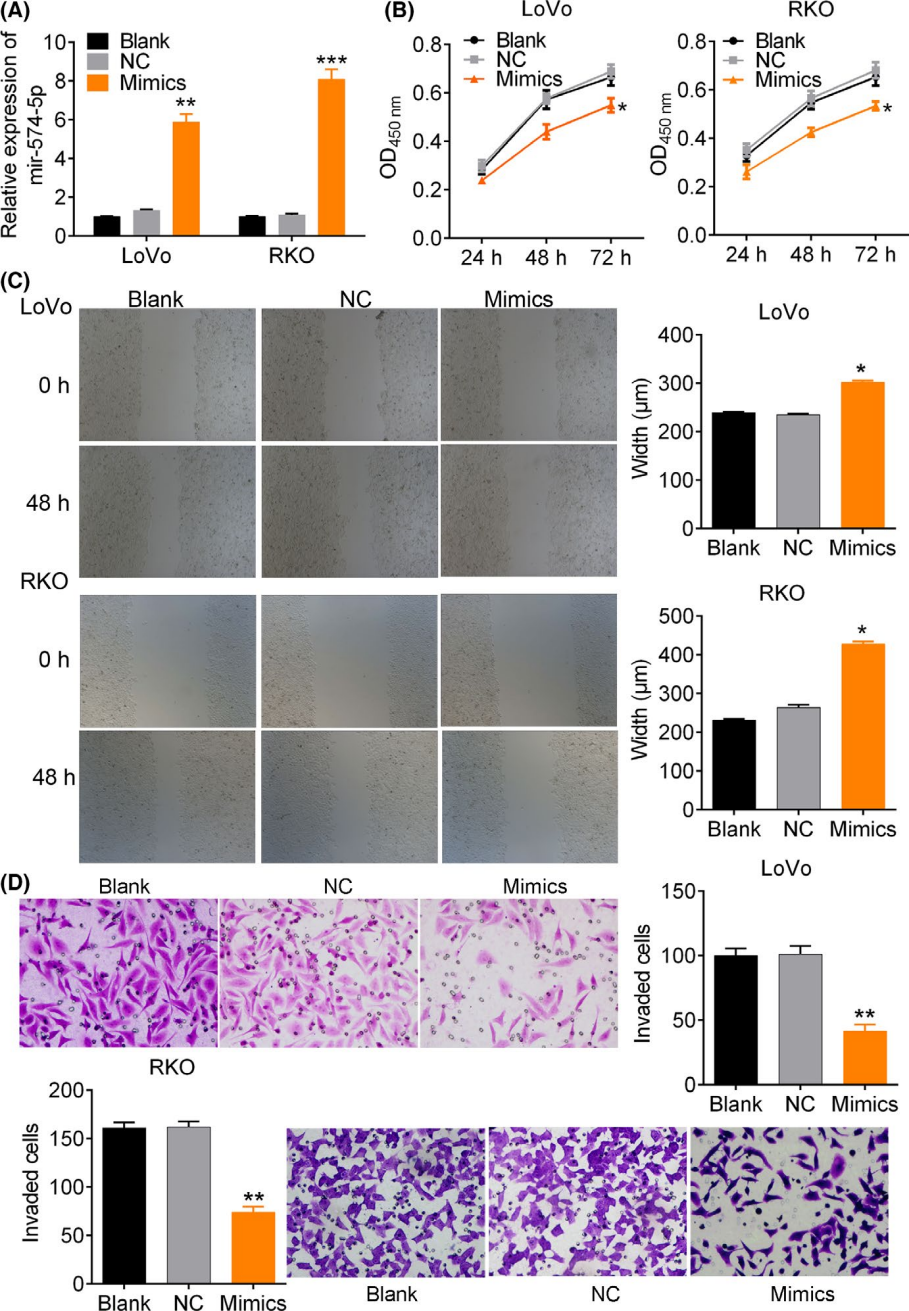
MiR‐574‐5p suppresses CRC cell proliferation and metastasis. A, the relative expression of miRNA in cells transfected with miR‐574‐5p mimics. B, cell viability analysis by CCK‐8 assay. C, wound healing assay. Cells were transfected with miR‐574‐5p mimics for 48 h. D, cell invasion assay by transwell assay. Magnification, ×400. *, ** and *** note *P* < 0.05, *P* < 0.01 and *P* < 0.001 vs NC, respectively. NC, negative control

**Figure 6:**
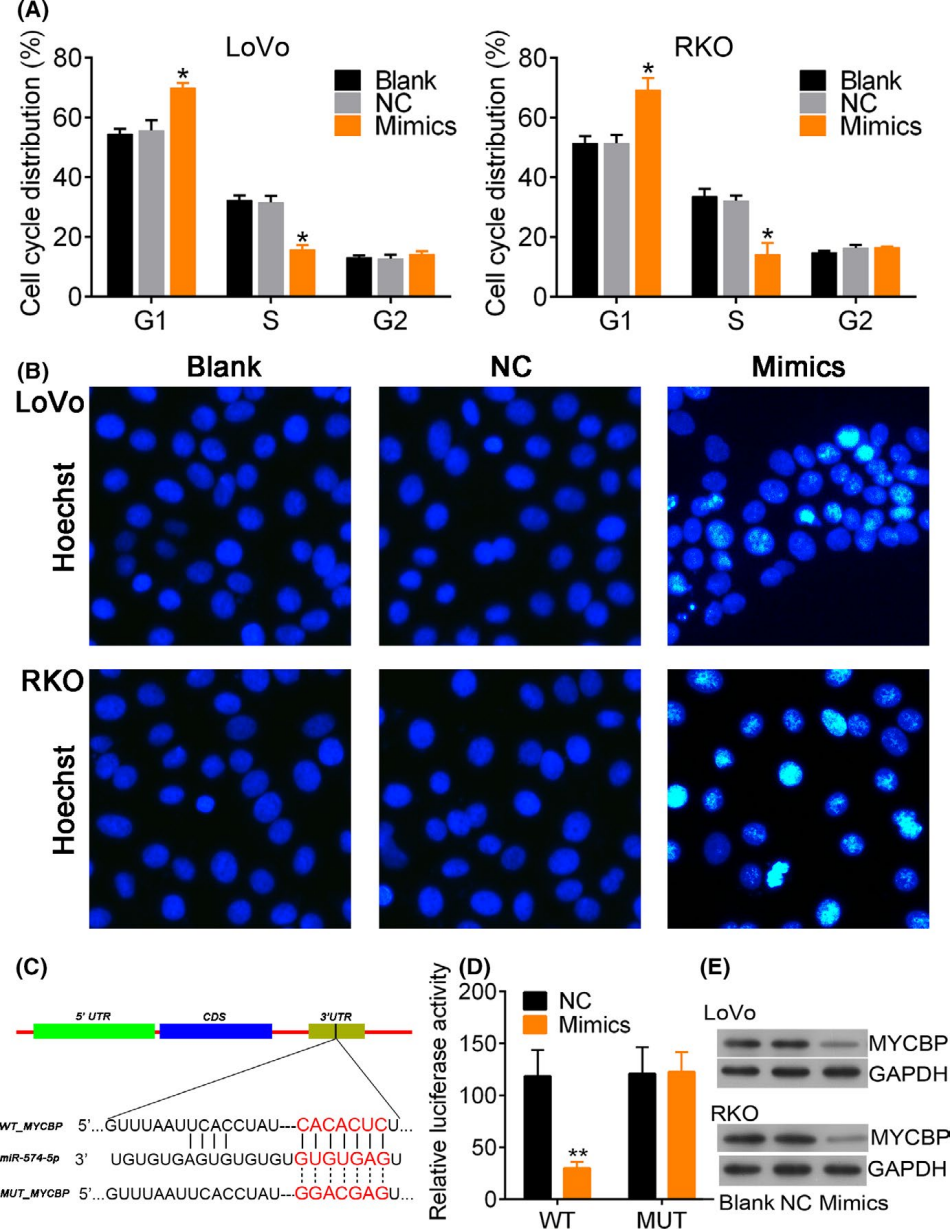
MFI2‐AS1 siRNA affects cell cycle distribution and apoptosis. A, Cell cycle analysis was performed using flow cytometry. Cells were transfected with miR‐574‐5p mimics for 48 h. B, Hoechst staining for cells in response to miR‐574‐5p mimics. C, the predicated binds sites of miR‐574‐5p to MYCBP 3′‐UTR. D dual‐luciferase reporter assay. E, the western blot analysis of MYCBP protein in cells transfected with miR‐574‐5p mimics. * and ** note *P* < 0.05 and *P* < 0.01 vs NC, respectively. MUT, mutant 3′UTR of_MYCBP; NC, negative control; WT, wild type of 3′UTR

Figure 5 showed that the miR‐574‐5p expression enhanced by mimics (Figure 5A) dramatically inhibited cell viability (*P* < 0.05, Figure 5B), cell migration (*P* < 0.05, Figure 5C) and invasion (*P* < 0.01, Figure 5D) compared to blank control. Flow cytometry analysis showed that miR‐574‐5p expression obviously up‐regulated the percentage of cells at G1 phase and reduced the cells at S phase (*P* < 0.05, Figure 6A and Figure S3), and enhanced DNA damage (Figure 6B). We then predicted that MYCBP, QKI, MACC1 and PTPRU were all the target genes of miR‐574‐5p by TargetScan Human. MYCBP expression was changed more significantly after miR‐574‐5p depletion, and so we predicted MYCBP as a target of miR‐574‐5p (Figure 6C, 122 ~ 128 nt). Dual‐luciferase reporter assay showed that the administration of miR‐574‐5p mimics significantly reduced the relative luciferase intensity (*P* < 0.01, Figure 6D). Moreover, we found that the expression of MYCBP protein was reduced by miR‐574‐5p mimics in LoVo and RKO cells (Figure 6E). These data suggested that miR‐574‐5p expression might suppress CRC cell proliferation and invasion via MYCBP expression inhibition.

### 3.5 MFI2‐AS1 regulates CRC cell proliferation and metastasis and MYCBP expression by sponging miR‐574‐5p

**Figure 7:**
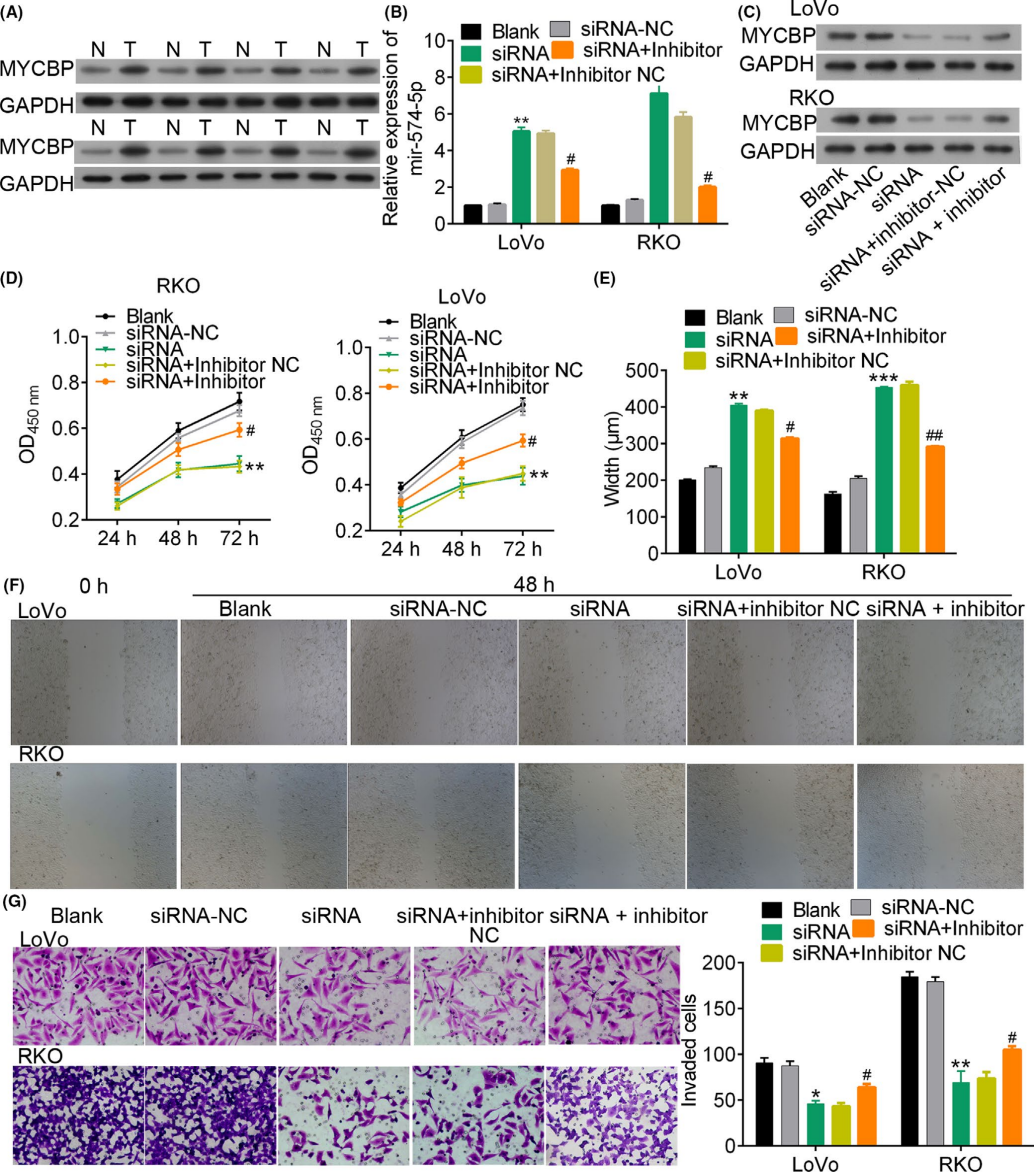
MiR‐574‐5p depletion rescues cell proliferation suppressed by MFI2‐AS1 siRNAs. A, western blotting of MYCBP protein levels in 8 pairs of CRC tumours and non‐tumour tissues. B and C, relative expression level of miRNA and MYCBP protein in cells and different transfection conditions, respectively. D, cell viability by CCK‐8 assay. E and F, wound healing assay of cells treated with different conditions for 48 h. G, invasion analysis by transwell migration assay. Magnification, ×400. *** and ### indicate *P* < 0.001 vs NC and siRNA, respectively. NC, negative control

**Figure 8:**
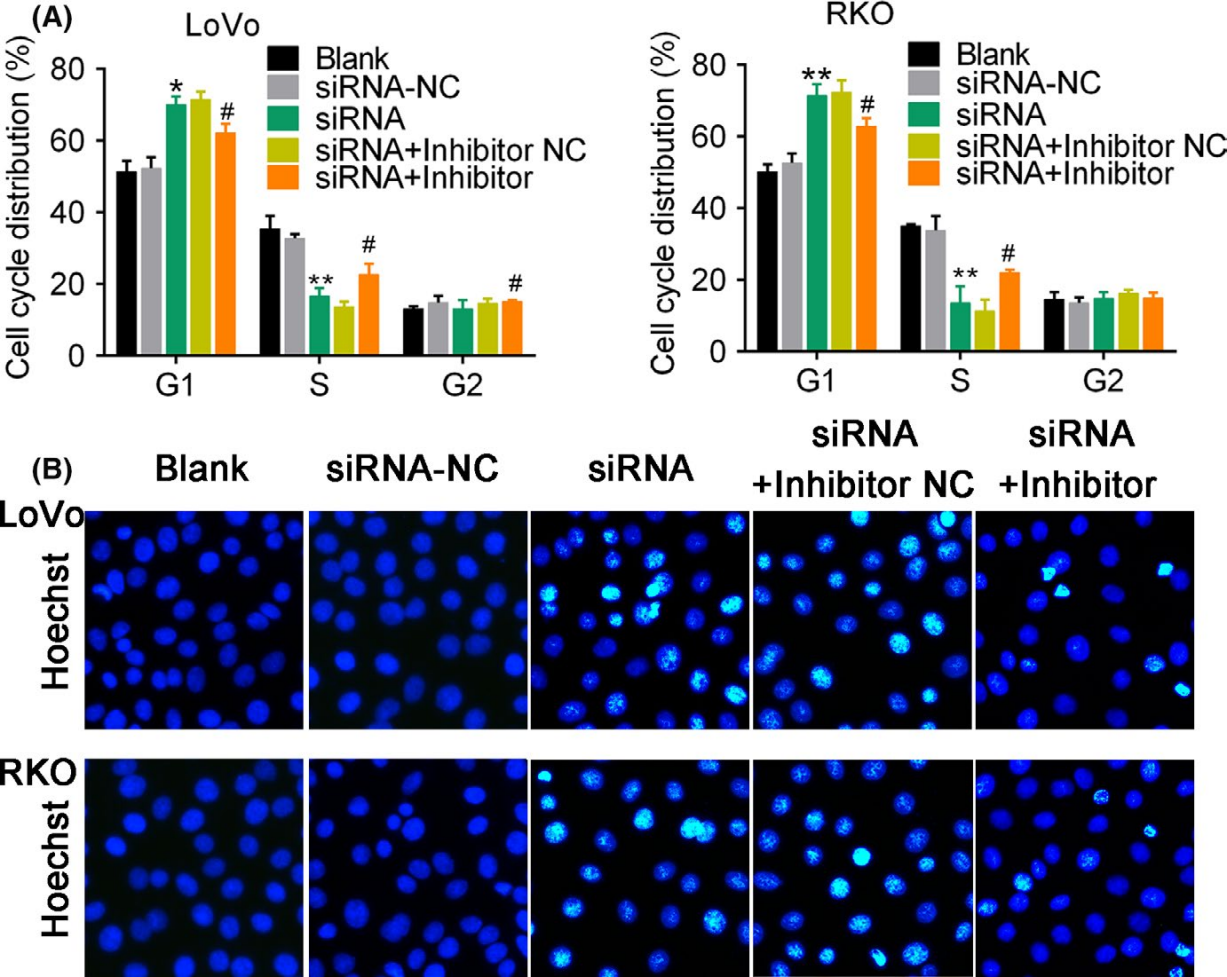
MiR‐574‐5p inhibition rescues MFI2‐AS1 siRNA‐influenced cell cycle distribution, apoptosis and DNA damage. A, Cell cycle analysis was performed using flow cytometry. Cells were transfected with miR‐574‐5p mimics for 48 h. B, Hoechst staining for cells in response to miR‐574‐5p mimics and MFI‐AS1 siRNA. Cells were transfected with different conditions for 48 h. * and ** note *P* < 0.05 and *P* < 0.01 vs. Blank, respectively. # and ## note *P* < 0.05 and *P* < 0.01 vs siRNA, respectively. NC, negative control

According to the dual‐luciferase reporter assay results, we speculated that lncRNA might function as a ceRNA for MYCBP in regulating CRC cell proliferation. We determined the up‐regulated expression of MYCBP protein in CRC tumour tissues by comparing with adjacent tissues (Figure 7A). The MFI2‐AS1 siRNA‐induced expression of miR‐574‐5p in CRC cells was dramatically suppressed by administration of miR‐574‐5p inhibitor (*P* < 0.01, Figure 7B). In contrast, the decreased MYCBP protein by MFI2‐AS1 siRNA was up‐regulated by miR‐574‐5p inhibitor (Figure 7C). These results suggested that the expression of MYCBP was competitively regulated by miR‐574‐5p and lncRNA MFI2‐AS1. Further analysis showed that MFI2‐AS1 siRNA inhibited cell viability (*P* < 0.01, Figure 7D), migration (*P* < 0.01, Figure 7E,F) and invasion (*P* < 0.01, Figure 7G). The increased percentage of cells at G1 phase (*P* < 0.01, Figure 8A and Figure S4) and Hoechst 33258‐positive cells (Figure 8B) were significantly rescued by the administration of miR‐574‐5p inhibitor. These data revealed that the MFI2‐AS1 siRNA inhibited CRC cell proliferation and metastasis and MYCBP inhibition could be rescued by miR‐574‐5p inhibitor, suggesting that MFI2‐AS1 promoted CRC metastasis via sponging miR‐574‐5p.

## 4 DISCUSSION

Numerous genetic factors including dysregulation of miRNAs and lncRNA are involved in the pathogenesis, development, metastasis and prognosis of CRC.^19^, ^20^, ^21^, ^30^, ^31^ MFI2‐AS1 is recently identified as a lncRNA, which is up‐regulated in pancreatic cancer cells and sporadic localized ccRCC.^24^, ^25^ Results of our study showed the up‐regulation of lncRNA MFI2‐AS1 in CRC tumour tissues compared with adjacent non‐tumour tissues. We further determined that the inhibition of MFI2‐AS1 inhibited proliferation, migration and invasion and promoted apoptosis by sponging miR‐574‐5p in CRC LoVo and RKO cell lines.

LncRNA MFI2‐AS1 is a novel lncRNA identified from drug‐resistant pancreatic cancer cells using next‐generation RNA sequencing.^24^ Flippot et al showed that the up‐regulation of MFI2‐AS1 was associated with poor survival of patients with sporadic localized ccRCC, and patients with undetectable MFI2‐AS1 had favourable outcomes.^25^ We identified that the expression of MFI2‐AS1 was significantly up‐regulated in CRC tumour tissues when compared with adjacent non‐tumour tissues. Bioinformatics analysis showed that MFI2‐AS1 expression was significantly associated with prognosis of CRC, and patients with high expression of MFI2‐AS1 have a shorter DFS and OS. Although we did not conduct prognostic analysis because of short follow‐up time, we indeed found a significant association between MFI2‐AS1 expression and patients' clinicopathologic factors, including histological grade, TNM stage, vascular invasion and so on, which are acknowledged as adverse prognostic factors. So, we suggested that MFI2‐AS1 might be associated with the prognosis of CRC.

To investigate the association of MFI2‐AS1 in CRC pathogenesis and metastasis in vitro, we inhibited the expression of it in CRC cell lines LoVo and RKO by siRNA. Results showed that the inhibition of MFI2‐AS1 inhibited cell proliferation, migration and invasion, and promoted cell apoptosis and DNA damage by arresting cell cycle at G1 phase. These results demonstrated that the expression of MFI2‐AS1 was associated with the migratory and invasive abilities of CRC cells in vitro.

Bioinformatics analysis and the following experiment showed that MFI2‐AS1 directly targets to miR‐574‐5p by binding to 3′‐UTR sequences. Further experiments showed that miR‐574‐5p mimics can reduce the capability of proliferation, migration and invasion of CRC cells, suggesting that miR‐574‐5p might play a tumour‐suppressive role in CRC. Interestingly, similar findings were reported in CRC, where miR‐574‐5p negatively regulates MACC‐1 expression to suppress CRC liver metastasis.^32^ Besides, Wang et al^33^ revealed that the miR‐574‐5p was mediated by a novel lncRNA linc‐ZNF469‐3. Both the knockdown of linc‐ZNF469‐3 and over‐expression of miR‐574‐5p reduced the migratory and invasive ability of MDA‐MB‐231 and LM2‐4175 breast cancer cells by inhibiting *ZEB1*.^33^ However, MiR‐574‐5p is also identified as an oncogene in several cancers, including thyroid carcinoma^26^, ^27^ and CRC.^28^ Ji et al^28^ showed that miR‐574‐5p was significantly up‐regulated in CRC tissues in C57BL/6‐^Apcmin/+^ mice model. MiR‐574‐5p expression increased proliferation, migration and invasion of SW480 and CT26 cells, and vice versa when inhibited. Therefore, we suggested that miR‐574‐5p might express in different levels and was regulated by context‐specific signalling pathways, which require further research. Our present study showed that the miR‐574‐5p mimic‐induced proliferation inhibition, migration and invasion of CRC cells were associated with MYCBP. We found that both MYCBP and MFI2‐AS1 were up‐regulated in CRC tissues when compared with adjacent non‐tumour tissues. These data suggested the association of MFI2‐AS1/miR‐574‐5p/MYCBP axis with CRC pathogenesis and development.

MYCBP protein binds to proto‐oncogenes MYC to enhance the ability of c‐MYC protein‐promoted tumorigenesis.^34^, ^35^ The over‐expression of MYCBP promoted the invasion and migration of gastric cancer cells, and vice versa when inhibited.^35^ In addition, the MYCBP is a negative target of tumour‐suppressive miR‐22.^36^ Duan et al^37^ showed that the MYCBP is a target of tumour‐suppressive miR‐516b, and its down‐regulation promoted ameloblastoma cell apoptosis by inhibiting cell proliferation, migration and invasion through MYCBP/c‐myc/RECK/MMP signalling pathway. Both miR‐22 and miR‐516b are identified as tumour‐suppressive miRNAs^38^, ^39^, ^40^, ^41^ or oncogenic miRNAs.^42^, ^43^ These studies suggested the multifunctional roles of miRNAs in the pathogenesis.^42^ In the present study, the facts that both lncRNA MFI2‐AS1 and MYCBP were up‐regulated in CRC tumour tissues when compared with adjacent non‐tumour tissues might reveal the oncogenic roles of lncRNA MFI2‐AS1 and MYCBP in CRC pathogenesis and development. The facts that (a) MFI2‐AS1 sponged miR‐574‐5p and miR‐574‐5p were targeted to MYCBP; (b) miR‐574‐5p mimics inhibited proliferation, migration and invasion of CRC cells; and (c) miR‐574‐5p inhibitor retrieved the MFI2‐AS1 siRNA‐induced changes in CRC cells suggested the tumour suppressor role of miR‐574‐5p expression in CRC.

In conclusion, our study revealed that lncRNA MFI2‐AS1 and MYCBP were up‐regulated in CRC tumour tissues when compared with non‐tumour control tissues. SiRNA target MFI2‐AS1 and miR‐574‐5p mimics decreased cell proliferation, migration and invasion, and induced DNA damage and G1 phase arrest in LoVo and RKO cells. The target interaction between lncRNA MFI2‐AS1 and miR‐574‐5p as well as between miR‐574‐5p and MYCBP was detected using dual‐luciferase reporter assay. Taken together, our study suggested the facilitative role of lncRNA MFI2‐AS1 in CRC through MYCBP and by sponging miR‐574‐5p.

## CONFLICT OF INTEREST

Authors declared no conflict of interest.
