# Supplementary figures and images for: Non‐coding RNA MFI2‐AS1 promotes colorectal cancer cell proliferation, migration and invasion through miR‐574‐5p/MYCBP axis
Source: Cell Prolif. 2019 May 16;52(4):e12632. doi: 10.1111/cpr.12632 (PMC6668983; doi:10.1111/cpr.12632)

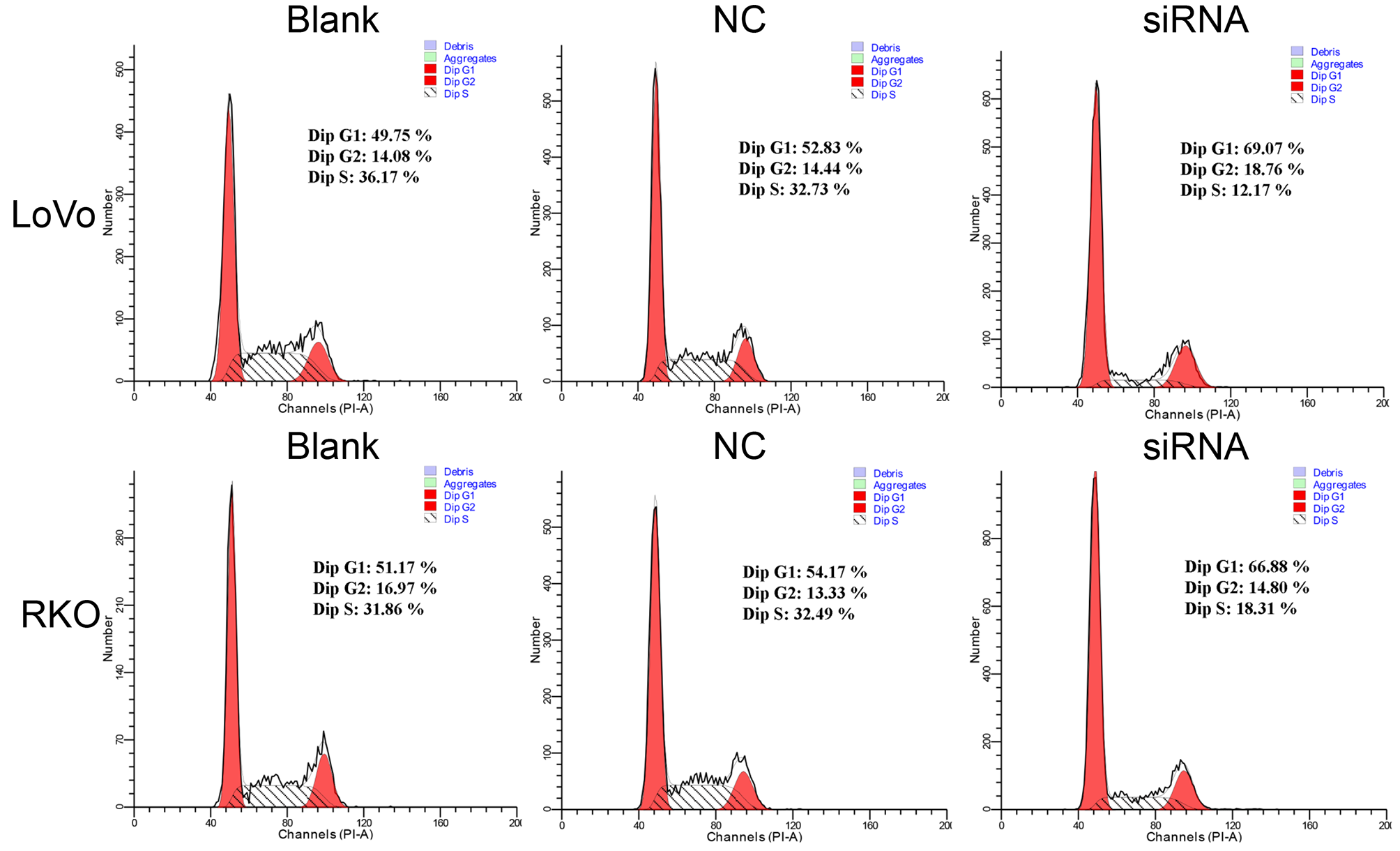

Supplement: Supplementary file 1 [file CPR-52-e12632-s001.tif]

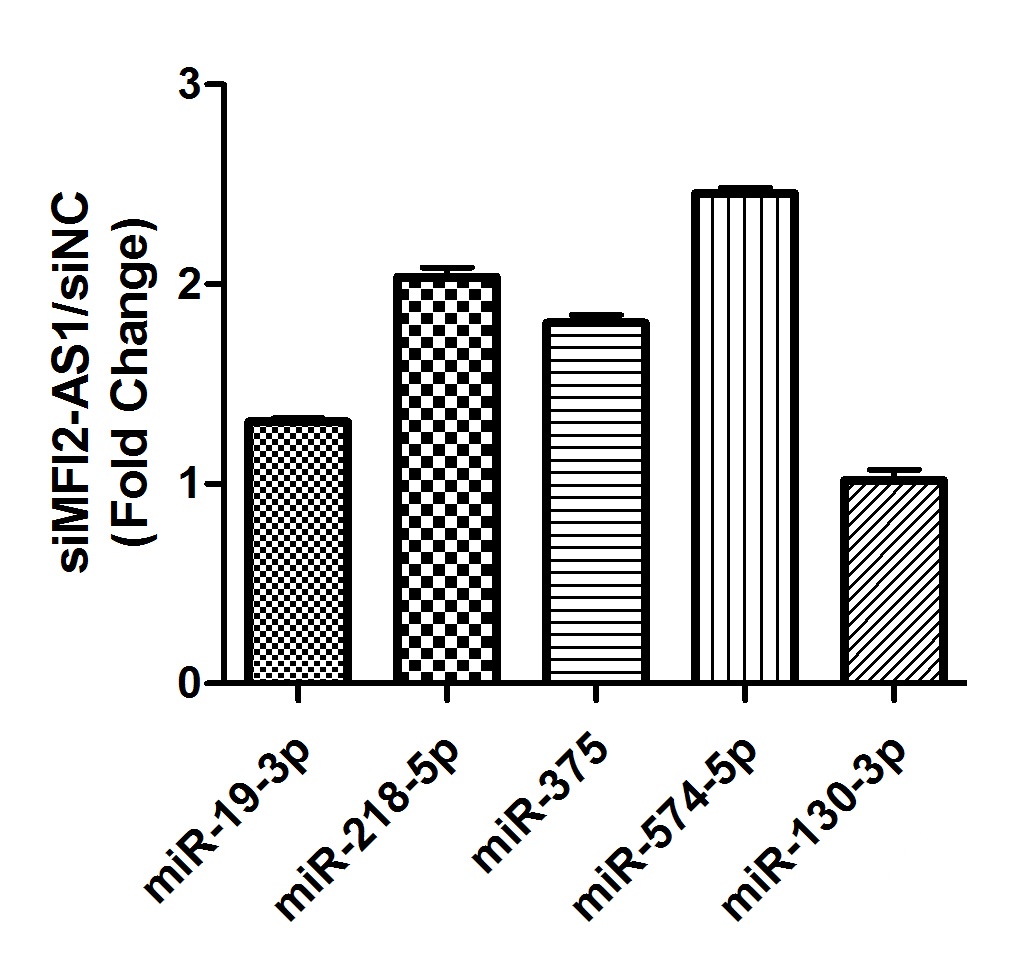

Supplement: Supplementary file 2 [file CPR-52-e12632-s002.jpg]

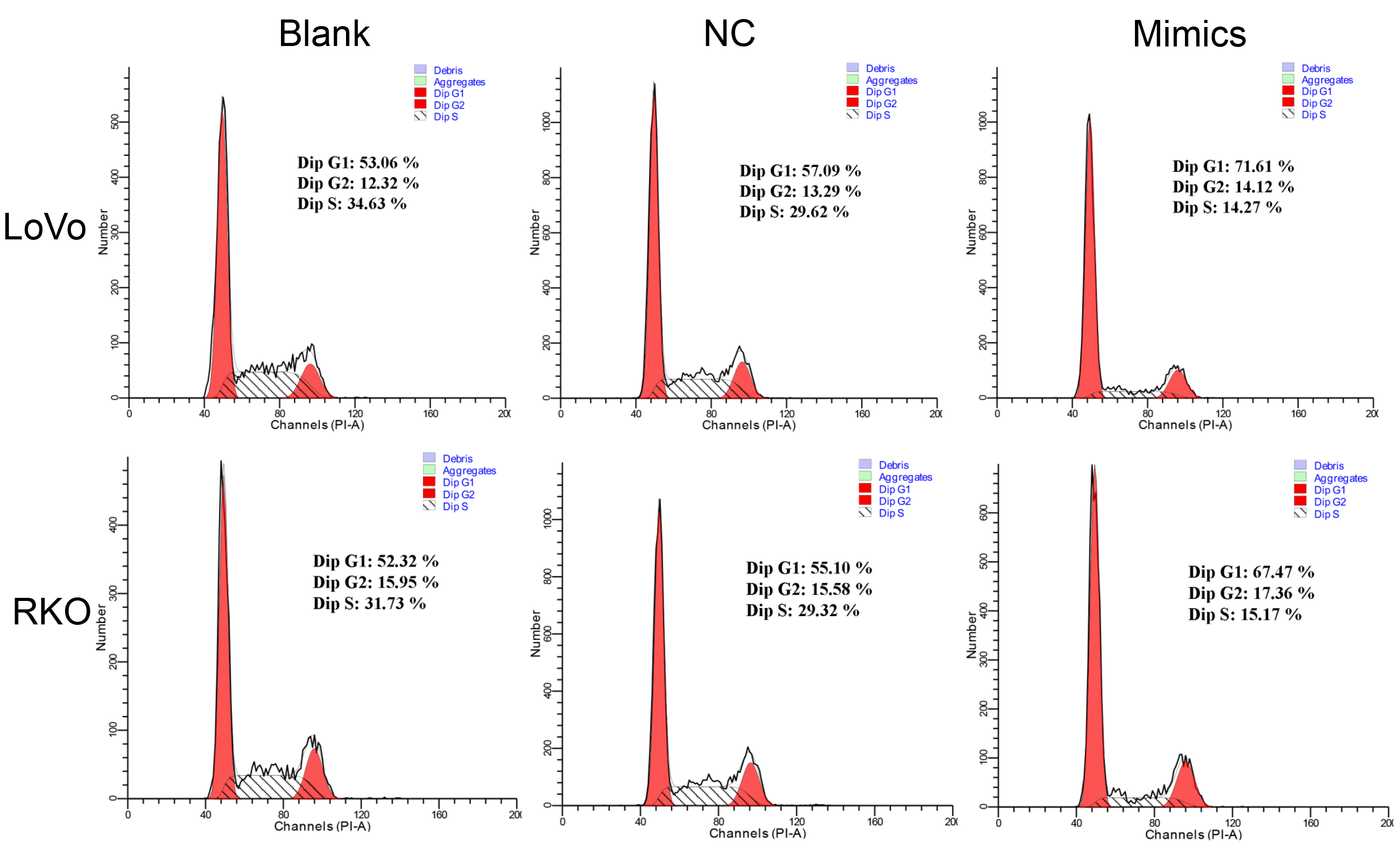

Supplement: Supplementary file 3 [file CPR-52-e12632-s003.tif]

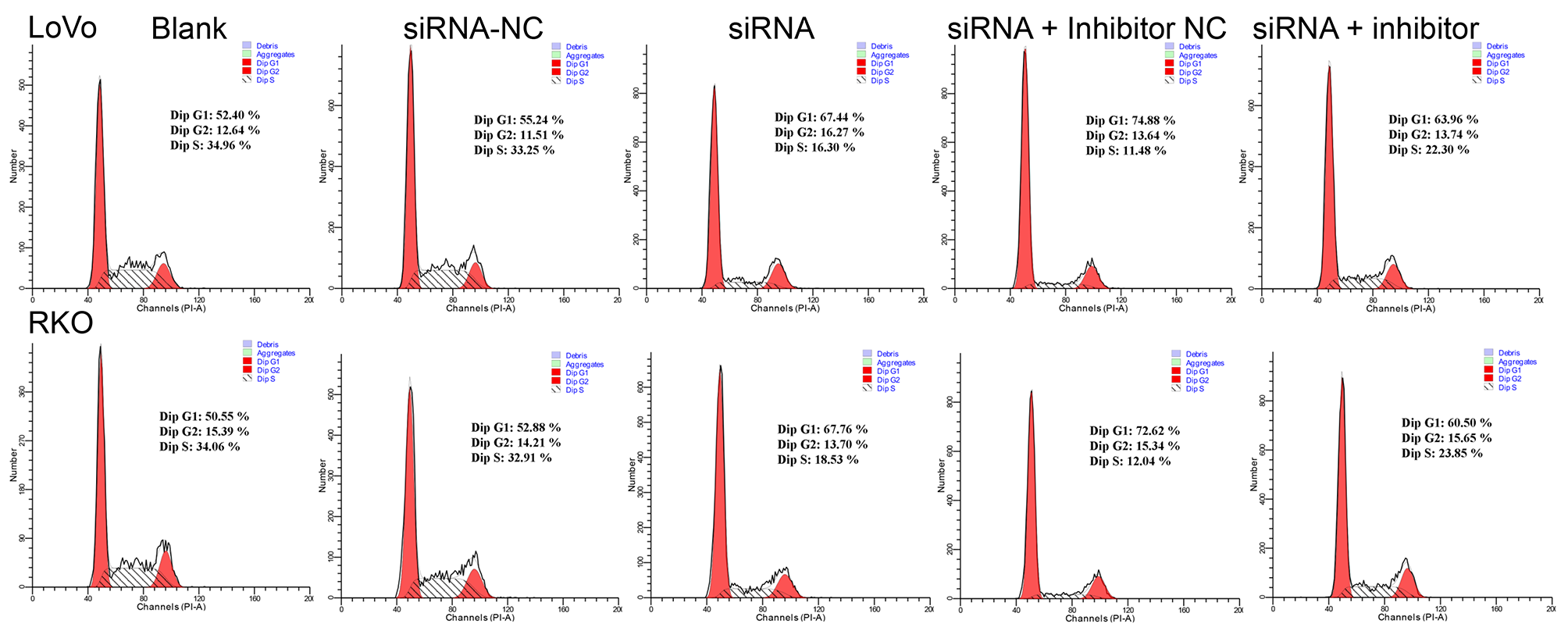

Supplement: Supplementary file 4 [file CPR-52-e12632-s004.tif]
